# A Rare Case of Concomitant Septic Arthritis, Osteomyelitis, and Pyomyositis Caused by Salmonella

**DOI:** 10.7759/cureus.20365

**Published:** 2021-12-12

**Authors:** Haider Ghazanfar, Iqra Nawaz, Ked Fortuzi, Arlene Tieng, Giovanni Franchin

**Affiliations:** 1 Internal Medicine, Bronxcare Health System, Bronx, USA; 2 Department of Internal Medicine/Rheumatology, Bronxcare Health System, Bronx, USA

**Keywords:** sickle cell anemia, pyomyositis, osteomyelitis, septic arthritis, salmonella typhi

## Abstract

A common causative organism in osteomyelitis in sickle cell disease is *Salmonella*. Septic arthritis and muscle infection due to *Salmonella* are much less common. We present a case of a 28-year-old woman with sickle cell disease who presented with left shoulder and elbow pain for two days. Physical examination revealed swelling of the left upper arm. The patient was initially treated for a sickle cell pain crisis. On hospital day 4, the patient developed a fever. She empirically started intravenous vancomycin and cefepime before her blood culture showed *Salmonella*. Subsequently, the antibiotic was changed to ceftriaxone. Synovial fluid analysis of the left shoulder revealed a white blood cell count of 53,250/mm^3^ with mostly neutrophils, and this led to a presumptive diagnosis of septic arthritis. She underwent a left shoulder arthroscopic irrigation and debridement. The synovial fluid culture was negative. Magnetic resonance imaging (MRI) revealed osteomyelitis in the left humerus, a 4.4 x 5 cm intramuscular abscess near the distal anterior humerus, and pyomyositis. Percutaneous abscess drainage was done. The patient was discharged home on ceftriaxone but returned 12 days later with worsening pain in her shoulder. Repeat MRI showed a complex glenohumeral joint effusion. She had an incision and drainage of her left shoulder. The patient was discharged on an eight-week course of ceftriaxone. Prompt diagnosis and early treatment are essential in reducing the mortality and morbidity associated with these joint, bone, and muscle infections.

## Introduction

Patients with sickle cell disease who present with acute pain in long bones without fever may be suspected of having vaso-occlusive pain. However, they should also be evaluated for other causes of pain that require treatment other than opioids. Patients with sickle cell disease are at increased risk for orthopedic complications such as osteonecrosis and bone infarction, but also infections such as osteomyelitis and septic arthritis [1]. Another rare infection that has been reported in sickle cell disease is pyomyositis [2]. Pyomyositis is an infection of the muscle that initially presents with pain, swelling, and fever, and then tenderness and edema.

The most common cause of pyomyositis and septic arthritis in adults is Staphylococcus aureus. However, *Salmonella* is more common in sickle cell disease pediatric patients with osteomyelitis in the United States [3]. It is unusual to see nontyphoidal Salmonellae in joint, bone, and muscle infections since this bacterial organism is known to cause diarrheal illness. We present this case of an adult patient with sickle cell disease with *Salmonella bacteremia* complicated by the shoulder and elbow septic arthritis, humeral osteomyelitis, and pyomyositis.

## Case presentation

**Figure 1:**
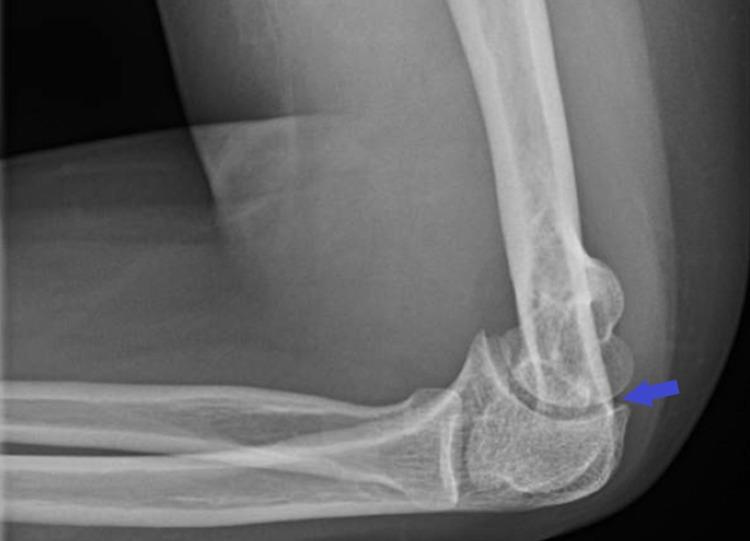
Radiography of the left elbow showing joint effusion (blue arrow)

A 28-year-old woman with sickle cell disease presented to the emergency department with pain in her left shoulder and elbow for two days. There was no preceding trauma and no fever, cough, nausea, vomiting, or diarrhea. Oral ibuprofen and morphine provided minimal pain relief. The patient had been admitted 14 days prior because of a sickle cell crisis with pneumonia and a urinary tract infection. She lived with her family and was not an injection drug user. On physical examination, the temperature was 98.2°F, the blood pressure 132/92 mmHg, the pulse was 97 beats per minute, the respiratory rate was 17 breaths per minute, and the oxygen saturation was 100% while the patient was breathing ambient air. Her left upper arm was mildly swollen and tender. Passive and active range of motion of the left shoulder and elbow was limited. Radiography of the left shoulder showed no evidence of acute fracture, and radiography of the left elbow showed joint effusion (Figure 1).

Laboratory test results are shown in Table 1.

**Table 1:** Initial laboratory test results

Investigation	Value	Reference Range
White blood cell (k/µL)	14	4.8-10.8
Hemoglobin (g/dL)	10.9	12.0-16.0
Hematocrit (%)	33.2	42.0-51.0
Platelet (k/µL)	557	150-440
Sodium (mEq/L)	136	135-145
Potassium (mEq/L)	4.4	3.5-50
Bicarbonate (mEq/L)	22	24-30
Chloride (mEq/L)	101	98-108
Glucose (mg/dL)	95	70-120
Blood urea nitrogen (mg/dL)	7	70-120
Creatinine (mg/dL)	0.7	8-26
Calcium (mg/dL)	9.7	0.5-1.5
Human immunodeficiency virus antibody	Negative	Negative

She was started on intravenous (IV) fluid and analgesia as needed. On day 4 of admission, she had a temperature of 102.3°F and was started on cefepime and IV vancomycin. Blood culture showed growth of *Salmonella *species, and the antibiotics were changed to ceftriaxone based on reported sensitivities. An ultrasound of the left upper extremity was unremarkable. Magnetic resonance imaging (MRI) of the left upper extremity without contrast showed diffuse humeral marrow abnormality, a moderate to large shoulder joint effusion, and elbow joint effusion. She underwent left shoulder joint and elbow joint aspiration, which revealed 3 mL of bloody synovial fluid and 20 mL of bloody synovial fluid, respectively. Synovial fluid analysis is shown in Table 2.

**Table 2:** Synovial fluid analysis

Investigation	Result
Shoulder Joint Synovial Fluid Analysis	
Color	Red
Gross Appearance	Cloudy
Red Blood Cell count (mil cells/mm^3^)	15,900
White Blood Cell count (cells/mm^3^)	53,250
Segmented white blood cell count (%)	96
Lymphocyte white blood cell count (%)	4.0
Aerobic Culture	No growth
Elbow Joint Synovial Fluid Analysis	
Color	Red
Gross Appearance	Cloudy
Red Blood Cell count (mil cells/mm^3^)	34,375
White Blood Cell count (cells/mm^3^)	113
Segmented white blood cell count (%)	90.0
Lymphocyte white blood cell count (%)	8.0
Band Body white blood cell count (%)	1.0
Eosinophil white blood cell count (%)	1.0
Crystal Examination	Negative
Culture	No growth

**Figure 2:**
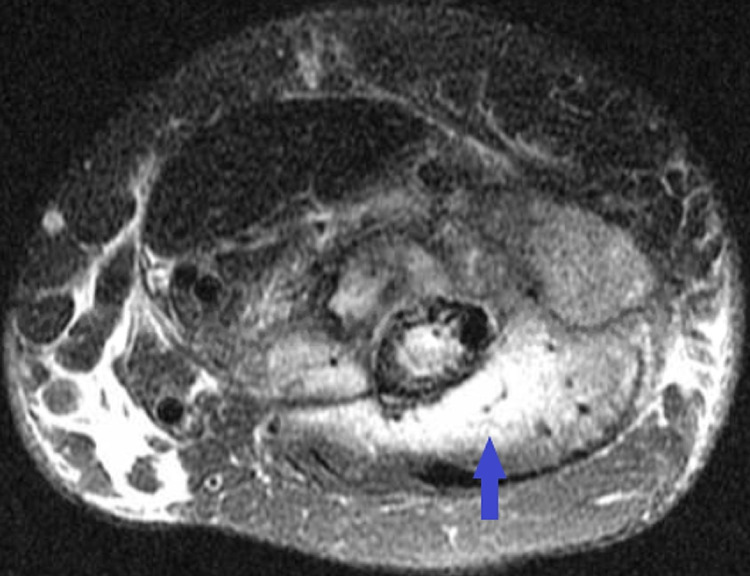
MRI of the left upper extremity showing showed left humerus and proximal radius osteomyelitis, myositis of brachialis, brachioradialis, and triceps muscles (blue arrow), complex glenohumeral joint effusion, and biceps tenosynovitis MRI: Magnetic Resonance Imaging

The patient underwent left shoulder arthroscopic irrigation and debridement. The synovial fluid culture showed no growth. Repeat MRI of the left upper extremity with contrast showed extensive osteomyelitis in the left humerus, a 1.7 x 4.4 x 5 cm intramuscular abscess near the distal anterior humerus, pyomyositis, and left glenohumeral and elbow joint effusions. The imaging-guided abscess drainage revealed 20 mL of turbid yellowish fluid with debris. Anaerobic, aerobic, and fungal cultures of the fluid showed no growth. On day 26 of this admission, she was discharged home on ceftriaxone and oral metronidazole for six weeks. However, she was re-admitted after 13 days due to progressive worsening of left upper extremity pain. Repeat MRI of the left upper extremity showed left humerus and proximal radius osteomyelitis, myositis of brachialis, brachioradialis, and triceps muscles, complex glenohumeral joint effusion, and biceps tenosynovitis (Figure 2).

A repeat ultrasound of the left upper extremity showed a small fluid collection measuring 11.6 x 6.6 mm in the left lateral aspect of the arm. A left shoulder arthroscopic incision and drainage were performed. Aerobic, anaerobic, and mycobacterial cultures of the aspirated fluid showed no growth. She was discharged home on an eight-week course of ceftriaxone. She was clinically stable at her follow-up appointment nearly two months later.

## Discussion

Musculoskeletal infection may not initially be suspected in a patient with sickle cell disease who presents with acute pain of an extremity. Differentiating between vaso-occlusive pain and joint, bone, and muscle infections tremendously affects management as antibiotics and surgical intervention may be required. Our patient presented with unilateral upper extremity pain without fever. Her restricted range of motion of shoulder and elbow joints implied that at least an oligoarthritis was involved. When she developed a fever, septic arthritis was immediately considered. 

*Salmonella* is known to cause osteomyelitis in sickle cell disease patients. It has also been reported to cause pyomyositis and septic arthritis. Septic arthritis and pyomyositis can be seen in IV drug users but our patient did not have this risk factor. Her synovial fluid culture may have been negative for *Salmonella* because the synovial fluid was obtained after a week of antibiotic use. Her shoulder infection was severe since it required arthroscopy twice. The sensitivity of organism identification in synovial fluid may be lower than in blood.

In addition to surgery, our patient was administered ceftriaxone to treat septic arthritis, osteomyelitis, and pyomyositis. There are no randomized controlled trials on the efficacy of the optimum antibiotic treatment approach for osteomyelitis in patients with sickle cell disease [4]. No bone biopsy for an organism was pursued since the blood culture showed growth of *Salmonella*. The pyomyositis was detected on the MRI after the positive blood culture and arthrocentesis. If the pyomyositis was detected first, vancomycin would have been the empiric antibiotic of choice [5]. There was no need to monitor the creatine kinase since her myositis was infectious, not inflammatory.

## Conclusions

This was an atypical clinical presentation of *Salmonella* infection as septic arthritis, osteomyelitis, and pyomyositis in sickle cell disease. Clinicians should consider the possibility of musculoskeletal infections in any sickle cell disease patient presenting with fever, muscle, bone, and joint pains. Prompt diagnosis and early treatment with IV antibiotics and surgery are essential in reducing the mortality and morbidity associated with joint, bone, and muscle infections.
